# Where Have All the FLOWERS Gone? A Multicenter Investigation of Frequent Users of Midwest Emergency Department Services During the COVID-19 Stay-at-home Orders

**DOI:** 10.5811/westjem.2022.7.55727

**Published:** 2022-09-15

**Authors:** Howard W. Levitin, Bruce G. Jones, Marie M. Lockhart, Christopher M. Lloyd, Meenal D. Sharkey, Paul A. Willette, Andrew F. Kalnow

**Affiliations:** OhioHealth Doctors Hospital, Department of Emergency Medicine, Columbus, Ohio

## Abstract

**Introduction:**

In this study we aimed to determine the impact of the mandatory coronavirus disease 2019 (COVID-19) pandemic stay-at-home order on the proportional makeup of emergency department (ED) visits by frequent users and super users.

**Methods:**

We conducted a secondary analysis of existing data using a multisite review of the medical records of 280,053 patients to measure the impact of the COVID-19 pandemic stay-at-home order on ED visits. The primary outcomes included analysis before and during the lockdown in determining ED use and unique characteristics of non-frequent, frequent, and super users of emergency services.

**Results:**

During the mandatory COVID-19 stay-at-home order (lockdown), the percentage of frequent users increased from 7.8% (pre-lockdown) to 21.8%. Super users increased from 0.7% to 4.7%, while non-frequent users dropped from 91.5% to 73.4%. Frequent users comprised 23.7% of all visits (4% increase), while super user encounters (4.7%) increased by 53%. Patients who used Medicaid and Medicare increased by 39.3% and 4.6%, respectively, while those who were uninsured increased ED use by 190.3% during the lockdown.

**Conclusion:**

When barriers to accessing healthcare are implemented as part of a broader measure to reduce the spread of an infectious agent, individuals reliant on these services are more likely to seek out the ED for their medical needs. Policymakers considering future pandemic planning should consider this finding to ensure that vital healthcare resources are allocated appropriately.

## INTRODUCTION

### Background

On March 13, 2020, the United States issued a National Emergency Declaration to slow the spread of the severe acute respiratory syndrome coronavirus 2 (SARS-CoV-2), the causative agent of the infectious coronavirus disease 2019 (COVID-19). Most state governors executed this declaration by implementing stay-at-home orders designed to limit people’s movement in public and reduce viral spread.^1^ These orders, directly and indirectly, resulted in a 42% reduction in emergency department (ED) visits nationally for a broad range of medical conditions and patient concerns.^2^–^6^

What is unknown about the lockdown is whether the decrease in ED visits was uniform across all patient demographics or whether there were specific subgroups, such as frequent ED users (FEDU), whose habits deviated from this trend. Frequent ED users are patients who historically consume a significant percentage of acute care resources.^7^,^8^ In general, these individuals have four or more ED visits per year, are more likely to suffer from three or more chronic medical conditions, have a higher incidence of mental health problems and substance use disorder, and account for a disproportionate amount of healthcare costs.^9^–^12^ The FEDU tend to be socioeconomically disadvantaged and have higher usage rates of outpatient offerings (eg, social work services, addiction treatment, psychiatric counseling) than non-frequent ED users (NFEDU).^10^,^11^ Overall, persons who seek out acute care more than four times per year represent between 3.5–29% of all ED patients but constitute 12.1–67% of all ED visits made.^12^

A subset of frequent users visits the ED 10 or more times per year. These ED super users (EDSU) account for only 2.6–6.1% of all ED patients but comprise 16.2% of Medicare patients (≥65 years), 26.2% of Medicare patients (age 1 to 64 years), 16.7% of Medicaid patients, and 10.5% of those patients with private insurance. Only 3.7% of all Medicaid ED patients were super users, but they accounted for more than five times the average ED charge.^12^

The **f**requent utilization **o**f midwest **e**mergency **r**oom **s**ervices (**FLOWERS**) study is a retrospective analysis of the effects of the stay-at-home order on the use of ED services by FEDU and EDSU during the early phase of the COVID-19 pandemic. This investigation included 20 EDs with diverse demographics, economic bases, and hospital types (eg, tertiary referral hospitals, trauma centers, academic and community hospitals, and freestanding EDs).

### Importance

Barriers to accessing healthcare services more commonly affect the impoverished, children, those with chronic illnesses, immigrants, the uninsured, and those with psychiatric and substance abuse disorders. Such barriers often lead to poorer health outcomes.^13^ When access to primary medical care is limited or reduced, patients commonly seek out services in the ED.^14^,^15^ The COVID-19 stay-at-home order was an emergency public health measure implemented in Ohio to help reduce community spread of disease during the pandemic. This measure created a broad, temporary barrier to healthcare access and a unique opportunity to assess its impact on at-risk patients who frequently use the ED for their healthcare needs.

The COVID-19 pandemic resulted in a marked drop in ED visits throughout the US and most of the world.^2^ This reduction occurred against a backdrop of rising morbidity and mortality from COVID-19 and untreated medical emergencies. How individuals choose to address their medical needs and concerns during a government-imposed lockdown has broad implications for the healthcare market and mode of medical service delivery. It also influences the focus of emergency management and public health planning, resource allocation, and community support assistance in the future.

**Population Health Research Capsule**

What do we already know about this issue?

*COVID stay-at-home orders implemented by state governors contributed to a 42% reduction in ED visits nationally for a broad range of medical conditions and patient concerns.*

What was the research question?

*Was the decrease in ED visits during the COVID-19 lockdown uniform across all patient demographics?*

What was the major finding of the study?

*During the COVID-19 lockdown the percentage of ED visits by frequent users increased by 179% while visits by super users increased by 571%.*

How does this improve population health?

*Policymakers need to understand the impact on individuals’ mental and physical health when they are discouraged from seeking medical care during an infectious disease outbreak.*

### Goals of This Investigation

Our primary goal in this investigation was to determine the impact of the COVID-19 pandemic stay-at-home order on use of the ED by people who were FEDUs and EDSUs prior to the pandemic lockdown. We hypothesized that FEDUs and EDSUs would increase their ED use during the emergency declaration. Our secondary outcomes were to determine whether unique patient demographics and encounter characteristics differed between non-frequent, frequent, and super users of ED services before and during the stay-at-home order.

## METHODS

### Study Design and Setting

The FLOWERS study was a secondary analysis of existing data for ED visits to a charitable Midwest healthcare system. This not-for-profit system has a network of 20 EDs (hospital-based, including a Level I and Level II trauma center and freestanding EDs) spanning 47 counties in Ohio with a combined annual ED census of 492,650 visits by patients ≥18 years of age. The study was conducted with the approval of the hospital’s institutional review board. The interval for the analysis included a designated 12-month period before and a 9-week interval during the mandatory COVID-19 stay-at-home order. Ohio’s stay-at-home order was implemented on March 23, 2020. The order included ceasing operation of all non-essential business, prohibition of all public and private gatherings, limitation of travel, closure of schools, cancellation of elective medical procedures, and implementation of social-distancing measures. Therefore, March 23, 2020, was selected as the initial reference point to begin assessing the impact of this public health measure on peoples’ willingness to seek ED care during the beginning of the mandatory lockdown. The cancellation of the order on May 29, 2020, was the end date in the study because it represented a transition point between lockdown and the resumption of business activities.

**Figure 1:**
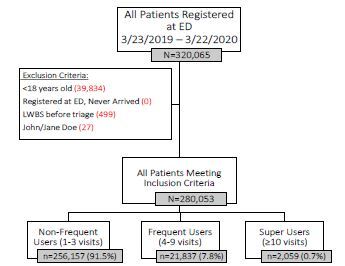
Flowchart outlining inclusion and exclusion criteria and methodology for assigning study participants into user groups. *ED*, emergency department; *LWBS*, left without being seen.

The purpose of this study was to determine the impact of the mandatory COVID-19 pandemic stay-at-home order on the proportional makeup of ED visits by FEDUs and EDSUs. We used the total number of ED visits each registered person made over the prior 12 months (March 23, 2019–March 22, 2020) to categorize each patient into one of three user groups (Figure 1). We then compared the proportional makeup of each group during the prior 12 months to the 9-week emergency declaration period (March 23–May 29, 2020).

The study compared patient and admission characteristics for both periods. This information included ethnicity, race, ED disposition, insurance status, and arrival means. During the ED registration process, each patient’s ethnicity was determined by inquiring whether they identified as being Hispanic or non-Hispanic. Patients were also asked about their race identification. Patients who self-identified as White or Caucasian were entered as the former, while the options Black or African American were entered as both. Patients who did not provide their race were given the following choices: White, African American or Black, American Indian or Alaska Native, Asian, Native Hawaiian or other Pacific Islander, or other designation (unknown, declined to specify, or two or more races). We collected race and ethnicity data to determine whether specific groups were impacted differently during the COVID-19 stay-at-home order.

### Selection of Participants

Subjects for this study included 280,053 eligible individuals who registered to be evaluated in any of the 20 designated EDs during a 12-month period (March 23, 2019–March 22, 2020). These patients were identified by an electronic health record (EHR) query performed by a trained data analyst in the Quality and Patient Services (QPS) Department. The study excluded participants if they were <18 years of age, registered as John/Jane Does, were designated as a hospital transfer who never arrived at one of the study locations, left without being seen by a clinician, or were evaluated in an urgent care facility. The principal investigator and designated study staff reviewed a subset of records for inclusion/exclusion criteria to determine the accuracy of the QPS data query. The identified participants were subdivided into three groups based on their number of visits during the 12 months preceding the stay-at-home order: NFEDUs (<4 visits); FEDUs (4–9 visits); and SEDUs (≥10 visits). For each patient within the three groups, we compared their prior ED use to their ED visits during the Ohio stay-at-home declaration in response to COVID-19, a nine-week period from March 23–May 29, 2020.

### Measurements and Analysis

Trained hospital data analysts collected patient and admission level data via a system-level query of the EHR (Epic Systems Corporation, Verona, WI) (Table 1). The study team validated a subset of records using manual health records review to ensure data accuracy for each set of inclusion and exclusion criteria added to the query. Emergency department encounter characteristics of interest for this study included means of arrival, insurance status, and disposition, while patient-specific characteristics included gender, age, ethnicity, and race. We summarized all patient- and encounter-level data using means, percentages, and 95% confidence intervals. Missing data points were omitted from the calculation of percentages. For patients with repeat visits, we only reported data from a patient’s first encounter during each period (before and during the stay-at-home order). We analyzed all data with R version 4.1.1 (R Foundation for Statistical Computing, Vienna, Austria).^16^

### Outcomes

We examined four outcomes in an analysis of ED use before and during the mandatory COVID-19 stay-at-home lockdown: 1) proportion of NFEDUs (<3 annual ED visits); 2) proportion of FEDUs (4–9 annual ED visits); 3) proportion of EDSUs (≥10 annual ED visits); and 4) unique patient characteristics during the defined periods. These outcomes were chosen to determine whether the impact of the government-issued COVID-19 lockdown on ED visits were uniform across all patient demographics or whether there were specific subgroups whose ED use deviated from that of others.

## RESULTS

### Patient Emergency Department Use Groups

Figure 1 outlines the assignment of patients into three groups based on frequency of ED use. During this one-year period, 280,053 patients met the study criteria by registering for evaluation in one of 20 EDs within a single Midwestern healthcare system. Most patients (91.5%) were designated as NFEDU, while 7.8% and 0.7% were classified as FEDU and EDSU, respectively.

### Patient Demographics and Encounter Characteristics Pre-lockdown

A summary and comparison of demographic data from each group for the one-year preceding the COVID-19 lockdown period (ie, pre-lockdown) is presented in Table 2. In general, patient demographics between the groups were similar. Most of the ED patients in each group were female (55–62%), White (approximately 70%), and of a similar age range, with over 92% self-identified as neither Hispanic nor Latino. Those of Black descent had a slightly higher representation among FEDU and EDSU patients (25.6% and 27%, respectively) than the NFEDU patients (20.2%). Asian Americans demonstrated a corresponding reduction in the two groups (FEDU, 1.3%; EDSU, 0.9%; NFEDU, 1.9%).

Government-sponsored programs (Medicaid and Medicare) provided health insurance coverage to 52% of NFEDUs, 74% of FEDUs, and 83% of EDSUs. Individually, Medicaid use was less prevalent in NFEDUs (26.5%) than FEDUs and EDSUs (45.2% and 52.6%, respectively), while the use of Medicare among all groups remained steady (25.6–30.7%). Private insurance was used by 40.5% of FEDUs, 18% of NFEDUs, and 9.8% of EDSUs. Compared to NFEDUs, FEDUs and EDSUs were more likely to be uninsured (2.8% vs 5.5% and 5.3%, respectively). In addition, EDSU patients were more likely to be hospitalized, leave against medical advice, leave without being seen after triage, and leave before final disposition. The EDSU patients arrived at the ED by ambulance more frequently than NFEDU and FEDU patients (26.8% vs 17.9% and 19.6%, respectively). The EDSU patients used public transportation to arrive at the ED more often than NFEDU and FEDU patients (3.4% vs 0.9% and 1.4%, respectively), and EDSU patients arrived by a personal vehicle less commonly than NFEDU and FEDU patients (65% vs 79.1% vs 76.1%, respectively).

### Pre-Lockdown vs Lockdown

To determine whether the percentage of patients in each usage group changed during the mandatory lockdown, we compared the proportional makeup of each group between the two time periods. We included only known users who previously registered for ED care in the prior 12 months in this data analysis. Throughout the mandatory COVID-19 lockdown, the percentage of registered patients previously identified as FEDUs climbed from 7.8% (pre-lockdown) to 21.8% during lockdown (a 179% increase). The number of EDSUs grew by 571% (0.7% to 4.7%), while the number of NFEDUs dropped from 91.5% to 73.4% (a 19.8% decrease) during the lockdown (Table 3).

In comparing the proportions of ED visits for each group, we found that NFEDUs comprised 66.8% of all visits (a 6% decrease), FEDUs comprised 23.7% of all visits (a 3.9% increase), while EDSU encounters (9.5%) increased by 53% (Table 4). Overall, the combination of FEDUs and EDSUs in the pre-lockdown period comprised 8.5% of registered ED patients yet constituted 29% of all ED encounters. These two combined groups accounted for 26.5% of ED patients and 33.2% of ED encounters during the lockdown.

We also compared demographics and patient-level encounter characteristics between the two periods (Tables 5 and 6). We saw a slight decrease in the proportion of male patients who registered for ED care during lockdown (41.4% vs 44%), with a corresponding increase in female registrants (58.6% vs 56%). During the lockdown, the percentage of patients who self-identified as White reduced from 70.4% to 67.7%, while those identifying as either African American or Black increased from 20.8% to 25.4%. Age and ethnicity remained relatively unchanged before and during the lockdown.

The proportion of individuals with private insurance dropped from 38.4% during pre-lockdown to 22% during lockdown (a decrease of 42.7%), while the percentage of uninsured ED visitors rose from 3.1% to 9% (an increase of 190.3%). The proportion of patients covered by Medicaid increased from 28.2% to 39.3% (an increase of 39.3%), while the proportion of Medicare users remained essentially unchanged. A greater proportion of patients arrived by ambulance during the lockdown than before the lockdown (22% vs 18%), while fewer were transported to the ED by private vehicle (73.2% vs 78.7%). Patient disposition varied little between the two periods except for a slight increase in the proportion of those who left against medical advice (1.4% vs 1.1%), those who were hospitalized (21.4% vs 19.4%), and those patients sent to labor and delivery (0.1% vs 0.04%) during the lockdown.

A total of 16,190 new patient encounters occurred during the lockdown. These ED visits were by individuals who had not previously registered for care during the prior year. As a result, these new patients were not included when calculating patient encounters and demographic data during the lockdown.

## DISCUSSION

In 1955, the legendary songwriter and folk singer Pete Seeger wrote “Where Have All the Flowers Gone?”—an anti-war song about how wars can destroy an entire generation of young people. In the lyrics, young girls picked flowers to put on their boyfriends’ graves, all of whom had died in battle. The song was translated into over 30 languages and helped define a generation.^18^ Like war, pandemics also impact generations of people. The spread of SARS-CoV-2 resulted in over 255 million cases of COVID-19, and five million deaths worldwide, including 47.5 million cases in the US and over 768,000 deaths as of November 18, 2021.^19^ The virus caused widespread economic hardships and exacerbated long-standing systemic health and social inequalities, placing individuals from racial and ethnic minorities at higher risk of getting sick and dying from COVID-19.^20^,^21^

To curb the spread of disease, most governors throughout the US implemented mandatory stay-at-home orders. Healthcare systems followed suit by canceling elective procedures and limiting clinic and private practice hours. These actions, combined with the practices of social distancing, remote working, business, and school closures, and diminished vehicular usage, likely contributed to the nearly 40% curtailment in ED visits throughout the country. This reduction in ED patient volume during the declared national emergency appeared to impact all demographics and led to a uniform drop in routine, non-COVID-19-related medical emergencies (eg, myocardial infarctions, strokes, chronic obstructive pulmonary disease exacerbations, and critical patient admissions).^3^–^6^,^22^ This dramatic change prompted our question: “Where have all the patients gone?”

The FLOWERS study was a large, multicenter, single-state, healthcare system retrospective investigation of the impact of the initial COVID-19 pandemic stay-at-home order on ED use by historically frequent users of emergency services. The objective was to determine whether the reduction in visits during this period was uniform for all patients, including those who historically are frequent users of ED services. We gathered data from 20 EDs (hospital-based and freestanding) spanning 47 counties, including 280,053 patients with 492,650 ED visits.

The FEDUs are often chronically ill individuals with several active comorbidities, socioeconomically disadvantaged, and high users of both ED and outpatient services. Any barrier to accessing routine medical care, such as the COVID-19 stay-at-home order, should have increased ED visits from all patient groups, especially among frequent users of these services. However, we found that while patients who historically used ED services frequently did so more often during the lockdown, patients who were not frequent users tended to use ED services even less during the lockdown. The increased use among FEDUs and EDSUs was likely due to the reduced availability of other healthcare options.

Public health measures to reduce the spread of SARS-Co-V-2 likely impacted healthcare systems’ ability to provide routine medical services such as disease screening, health maintenance therapy, and mental health counseling. These community-based mitigation efforts, combined with deferred and delayed presentations of non-pandemic-related illnesses and pathologies, had negative implications worldwide.^23^ According to the World Health Organization, 42% of countries had disruptions in cancer care, 49% had disruptions in diabetes care, and 31% for cardiovascular disease services during the early phase of the pandemic. These routine clinical services reductions likely contributed to excess deaths from treatable and preventable non-COVID-19-related health conditions and illnesses.^24^–^27^ In addition, barriers to accessing routine healthcare services were likely the impetus for frequent users to seek out the ED in more significant numbers during the COVID-19 lockdown. Suppose these barriers and regular healthcare avoidance behaviors continued because of ongoing infection spread. In that case, patients could likely miss opportunities for acute medical interventions and necessary ongoing management of chronic conditions, vaccinations, and early screening for new medical problems that could worsen outcomes.^25^

Delays or avoidance in seeking medical care might have also contributed to excess deaths during lockdown periods. A web-based survey conducted from June 24–30, 2020 estimated that 40.9% of US adults aged ≥18 years avoided care during the pandemic due to concerns over COVID-19. This forestalling included 12% who avoided urgent or emergency care and 31.5% who avoided routine care. Avoidance of urgent and emergent care was highest among unpaid adult caregivers, individuals with two or more underlying health conditions, persons with health insurance, Black and Hispanic patients, young adults, and persons with disabilities. Those falling under one or more of these categories also represent those who were at increased risk of developing severe COVID-19.^25^,^28^

As a corollary, the reduction in healthcare utilization during the mandatory stay-at-home order may have had an unintended net positive effect on an individual’s health. According to a physician survey conducted in 2017, an interpolated median of responses revealed that 20.6% of overall medical care might be unnecessary, including 22% of prescription medications, 24.9% of tests, and 11.1% of procedures. The top reasons cited for overtreatment included fear of malpractice (84.7%), patient pressure/request (59%), and difficulty assessing medical records (38.2%).^26^ Also, individuals with higher incomes tend to undergo more expensive and extensive cancer screening exams to detect smaller abnormalities that lead to more follow-up testing and biopsies with little to no impact on mortality.^29^ Additionally, as many as one-third of hospitalized patients may experience harm or an adverse event, often from preventable errors. In 2009, total excess costs in US healthcare exceeded $750 billion due to perceived unnecessary and inefficiently delivered services, excess administrative costs, too high prices, missed prevention opportunities, and fraud.^30^

Data gathered from this study will be incorporated into future efforts to assess the impact of confounding factors to determine whether the lockdown disproportionately impacted the morbidity and mortality of NFEDUs and FEDUs. This ongoing research will also focus on community resources, family support systems, telemedicine, or other self-help strategies that either group may have used during the lockdown as an alternative to seeking emergent medical care. Such information may be useful in addressing these patients’ needs in the future.

## LIMITATIONS

There were several potential limitations to our study. There may have been an over-reporting of return visits in patients who were registered in the ED but left before completing evaluation only to return at a later time. This action could have increased their visit count by one, and if this patient visit was repeated, the accumulative effect might have incorrectly shifted them into one of the higher use groups. There is also a possibility that return visits may have been artificially elevated for patients whom the treating clinician requested that they return for a scheduled re-evaluation. These visits are typically not patient-centric decisions, as they are often the result of shared decision-making between the patient and the clinician. In addition, the exclusion of unidentified patients (ie, John/Jane Doe) from the study may have impacted the dataset if their number of ED admissions were significant. These individuals were each assigned a unique health record number that could be used to identify them upon return to the ED. The EHR system used by our health system is routinely updated to combine duplicate charts. If the data pull was redone now, there may be fewer John/Jane Doe in the dataset due to correction of errors by chart compilers. In our original dataset, there were 13 patients with John/Jane Doe status. Therefore, their inclusion in the study would not have impacted the results.

Another potential limitation involves the applicability of the study results to health systems in other states and locales. The government-issued, COVID-19 stay-at-home orders were not coordinated at the national level, which created the potential for implementation and impact variability. Although this variability was inherent to the process, the order’s overall negative effect on patients’ access to health services was generally uniform throughout the US.^17^ For example, delaying elective procedures and organ transplants were a common patient surge management strategy deployed by most healthcare systems.^17^ As a result of the stay-at-home order, dental offices closed and, in many states, nearly 80% of non-COVID-19 clinical trials were stopped or interrupted, including 400 clinical trials involving more than 200,000 cancer patients.^17^ In addition, there was a marked reduction in preventive screening procedures (eg, colonoscopies, mammograms, and routine lab tests for the management of chronic disease) because of community mitigation measures during the early phase of the COVID-19 pandemic. 17

This study did not assess for confounding factors beyond the mandatory COVID-19 stay-at-home order that may have impacted ED use, such as access to telemedicine, treatment advice offered online, and access to clinics that may have played a role in reducing the number of ED visits evaluated in the study. In addition, the data collected did not address other factors that may have influenced a patient’s decision to seek repeated ED care, including limited access to health services in the evenings or on weekends and holidays, referral by a primary care physician or specialist, perceived quality of care, or insurance status. We hope to explore these confounding factors to assess their impact on patients’ decisions to seek emergency care during a public health emergency in future work. We suspect that the study’s large sample size mitigated the potential influence of these limitations.

## CONCLUSION

It is incumbent upon clinicians and public health officials to better understand the impact on individuals’ mental and physical health when society’s most vulnerable are discouraged from using needed medical services during an infectious disease outbreak. An important part of this realization is addressing the implications of temporary disruptions in access to medical care during considerable periods of disease transmission. When barriers to accessing healthcare are implemented as part of a broader measure to reduce the spread of an infectious agent, individuals reliant on these services are more likely to seek out the ED for their medical needs. Future pandemic planning should consider this finding to ensure that vital healthcare resources are allocated appropriately.

## Figures and Tables

**Table 1:** Data dictionary

Variable	Value
Ethnicity	Not Hispanic or Latino, Hispanic or Latino, Declined
Race	White, African American or Black, American Indian or Alaska Native, Asian, Native Hawaiian or other Pacific Islander, Other (including unknown, declined to specify, two or more races)
ED disposition	Discharge, Left before final disposition, Hospitalize transfer to another facility, Left against medical advice, Left without being seen after triage, Expired, Sent to labor and delivery
Insurance status	Private insurance (including motor vehicle, accident, commercial, marketplace exchange), Medicaid, Medicare, Not covered (including self pay and hospital charity), Other (including VA, incarcerations, worker’s compensation)
Means of arrival	Ambulance (including medical flight transport), Personal vehicle, Public transportation (including taxi), Other (police, wheelchair, other)

*ED*, emergency department; *VA*, Veterans Affairs.

**Table 2:** Emergency department usage groups.

Demographics	Non-Frequent n = 256,157	Frequent n = 21,837	Super n = 2,059
Gender, % (CI)			
Male	44.6 (44.4–44.8)	38.1 (37.5–38.7)	43 (40.9–45.1)
Female	55.4 (55.2–55.6)	61.9 (61.3–62.5)	57 (54.9–59.1)
Unknown	<0.01	0	0
Age in years, mean (CI)	46.9 (46.8–47.0)	46.4 (46.1–46.7)	45.9 (45.2–46.6)
Ethnicity, % (CI)			
Not Hispanic or Latino	91.8 (91.7–91.9)	95.1 (94.8–95.4)	96.5 (95.7–97.3)
Hispanic or Latino	3.9 (3.8–4.0)	3.2 (3.0–3.4)	3.1 (2.4–3.8)
Declined	4.3 (4.2–4.4)	1.7 (1.5–1.9)	0.5 (0.2–0.8)
Race, % (CI)			
White	70.6 (70.4–70.8)	68.6 (68.0–69.2)	69.3 (67.3–71.3)
African American or Black	20.2 (20.0–20.4)	25.6 (25.0–26.2)	27 (25.1–28.9)
American Indian or Alaska Native	0.2 (0.18–0.22)	0.2 (0.14–0.26)	0.2 (0.07–0.4)
Asian	1.9 (1.8–2.0)	1.3 (1.1–1.5)	0.9 (0.5–1.3)
Native Hawaiian or other PI	0.2 (0.18–0.22)	0.2 (0.14–0.26)	0.1 (−0.4–0.2)
Other	6.9 (6.8–7.0)	4.1 (3.8–4.4)	2.6 (1.9–3.3)
Insurance Status, % (CI)			
Private insurance	40.5 (40.3–40.7)	18.0 (17.4–18.5)	9.8 (8.5–11.1)
Medicaid	26.5 (26.3–26.7)	45.2 (44.5–45.8)	52.6 (50.4–54.8)
Medicare	25.6 (25.4–25.7)	29.2 (28.6–29.8)	30.7 (28.7–32.7)
Not covered	2.8 (2.7–2.9)	5.5 (5.2–5.8)	5.3 (4.3–6.2)
Other	4.7 (4.6–4.8)	2.1 (1.9–2.3)	1.7 (1.1–2.2)
Disposition, % (CI)			
AMA	1.1 (1.06-1-1.4)	1.6 (1.4–1.8)	2.2 (1.6–2.8)
Discharge	73.6 (73.4–73.8)	70.5 (69.9–71.1)	67.8 (65.8–69.8)
Expired	0.2 (0.18–0.22)	0	0
Hospitalize	19.3 (19.1–19.5)	21 (20.5–21.5)	23.1 (21.3–24.9)
Left before final disposition	0.8 (0.77–0.83)	1.3 (1.1–1.5)	1.5 (1.0–2.0)
LWBS after triage	0.3 (0.28–0.32)	0.5 (0.4–0.6)	0.7 (0.3–1.1)
Sent to L&D	0.1 (0.08–0.11)	<0.1	0
Transfer	4.7 (4.6–4.8)	5.1 (4.8–5.4)	4.7 (3.8–5.6)
Arrival, % (CI)			
Ambulance	17.9 (17.8–18.0)	19.6 (19.1–20.1)	26.8 (24.9–28.7)
Personal	79.1 (78.9–79.3)	76.1 (75.5–76.7)	65 (62.9–67.1)
Public	0.9 (0.86–0.94)	1.4 (1.2–1.6)	3.4 (2.6–4.2)
Other	2.2 (2.1–2.3)	2.9 (2.7–3.1)	4.8 (3.9–5.7)

*ED*, emergency department; *CI*, confidence interval; *AMA*, against medical advice; *LWBS*, left without being seen; *L&D*, labor and delivery; *PI*, Pacific Islander.

**Table 3:** Emergency department utilization groups pre-lockdown vs during lockdown.

ED Utilization Groups	Pre-Lockdown N = 280,053	Lockdown N = 24,242	% Change
Non-frequent users (n) % (CI)	256,157 91.5 (97.4–97.6)	17,795 73.4 (72.8–74)	−19.8%
Frequent users (n) % (CI)	21,837 7.8 (7.7–7.9)	5,288 21.8 (21.3–22.3)	+179%
Super users (n) % (CI)	2,059 0.7 (0.67–0.73)	1,159 4.7 (4.4–5.0)	+571%

*ED*, emergency department; *CI*, confidence interval.

**Table 4:** Proportion of total encounters by patient groups.

Encounters	Pre-Lockdown N = 492,650	Lockdown N = 49,188	% Change
All encounters (N)	492,650	49,188	
New/unknown (n)	-	16,190	
Returning (n)	-	32,988	
Non-frequent users (n) % (CI)	350,135 71.1 (70.9–71.2)	22,050 66.8 (66.3–67.3)	−6.0%
Frequent users (n) % (CI)	112,135 22.8 (22.6–22.9)	7,821 23.7 (23.2–24.1)	+3.9%
Super users (n) % (CI)	30,377 6.2 (6.1–6.3)	3,127 9.5 (9.1–9.8)	+53.2%

*CI*, confidence interval.

**Table 5:** Patient demographics pre-lockdown vs lockdown.

Demographics	Pre-lockdown N = 280,053	Lockdown N = 24,242	% Change
Gender, %(CI)			
Male	44 (43.8–44.2)	41.4 (40.8–42.0)	−5.9%
Female	56 (55.8–56.2)	58.6 (58.0–59.2)	4.6%
Age, mean (CI)	46.9 (46.8–46.9)	46.1 (45.9–46.3)	−1.7%
Ethnicity, %(CI)			
Not Hispanic or Latino	92.1 (92–92.2)	94.2 (93.9–94.4)	2.3%
Hispanic or Latino	3.8 (3.7–3.9)	3.6 (3.4–3.8)	−5.3%
Declined	4.1 (4.0–4.2)	2.2 (2.0–2.4)	−46.3%
Race, %(CI)			
White	70.4 (70.2–70.6)	67.7 (67.1–68.3)	−3.8%
African American or Black	20.8 (20.6–21)	25.4 (24.9–25.9)	22.1%
American Indian or Alaska Native	0.2 (0.18–0.22)	0.2 (0.14–0.26)	0
Asian	1.9 (1.8–2.0)	1.7 (1.5–1.9)	−10.5%
Native Hawaiian or other PI	0.2 (0.18–0.22)	0.1 (0.06–0.14)	−50%
Other	6.6 (6.5–6.7)	4.9 (4.6–5.2)	−25.8%

*CI*, confidence interval; *PI*, Pacific Islander.

**Table 6:** Patient-level encounter characteristics.

Encounter data	Pre-lockdown N = 280,053	Lockdown N = 24,242	% Change
Insurance Status, %(CI)			
Private insurance	38.4 (38.2–38.5)	22.0 (21.5–22.5)	−42.7%
Medicaid	28.2 (28.1–28.4)	39.3 (38.6–39.9)	39.3%
Medicare	25.9 (25.7–26.1)	27.1 (26.5–27.7)	4.6%
Not covered	3.1 (3.0–3.11)	9.0 (8.6–9.3)	190.3%
Other	4.4 (4.36–4.51)	2.7 (2.5–2.9)	−38.6%
Disposition, %(CI)			
AMA	1.1 (1.06–1.14)	1.4 (1.3–1.5)	27.3%
Discharged	73.4 (73.2–73.6)	71.4 (70.8–72.0)	−2.7%
Expired	0.2 (0.18–0.22)	0.2 (0.14–0.25)	0
Hospitalized	19.4 (19.3–19.5)	21.4 (20.9–21.9)	10.3%
Left before final disposition	0.8 (0.77–0.83)	0.5 (0.4–0.59)	−37.5%
LWBS after triage	0.3 (0.28–0.32)	0.2 (0.14–0.26)	−33.3
Sent to L&D	0.04 (0.033–0.047)	0.1 (0.06–0.14)	150%
Transferred	4.7 (4.6–4.8)	5 (4.7–5.3)	6.4%
Arrival, %(CI)			
Ambulance	18.1 (18.0–18.2)	22.2 (21.7–22.7)	22.7%
Personal	78.7 (78.5–78.9)	73.2 (72.6–73.8)	−7.0%
Public	0.9 (0.87–0.93)	1.4 (1.3–1.5)	55.6%
Other	2.3 (2.2–2.4)	3.2 (3.0–3.4)	39.1%

*CI*, confidence interval; *AMA*, against medical advice; *LWBS*, left without being seen; *L&D*, labor and delivery.
